# Resveratrol and Astaxanthin Protect Primary Human Nasal Epithelial Cells Cultured at an Air-liquid Interface from an Acute Oxidant Exposure

**DOI:** 10.33696/signaling.3.084

**Published:** 2022

**Authors:** Ayaho Yamamoto, Peter D. Sly, Nelufa Begum, Abrey J. Yeo, Emmanuelle Fantino

**Affiliations:** 1 Child Health Research Centre, The University of Queensland, South Brisbane, Queensland 4101, Australia; 2 The University of Queensland Centre for Clinical Research, Herston, Queensland 4029, Australia

**Keywords:** Oxidative stress, Airway epithelium, Air-liquid interface culture, Signaling pathways, Mitochondria, Antioxidant, Astaxanthin, Resveratrol

## Abstract

Oxidative stress (OS) in the airway epithelium is associated with cell damage, inflammation, and mitochondrial dysfunction that may initiate or worsen respiratory disease. However, it is unclear whether exogenous antioxidants can provide protection to the airway epithelium from OS. Resveratrol and astaxanthin are nutritional compounds that have shown diverse benefits including protection against OS and inflammation in various situations. The aim of this study was to examine the utility of pre-treatment with resveratrol and astaxanthin to prevent the negative effects of oxidant exposure and restore redox homeostasis in a well-differentiated epithelium grown from primary human nasal epithelial cells (NECs) at the air-liquid interface. Fully differentiated NECs were pretreated with the antioxidants for 24 hours and the cultured epithelia was subsequently exposed to hydrogen peroxide (H_2_O_2_) for 1 hour to induce an acute OS. Responses measured included mitochondrial reactive oxygen species (mtROS) generation, redox status (GSH/GSSG ratio), cellular ATP, and signaling pathways (SIRT1, FOXO3, p21, PINK1, PARKIN, NRF2). Following H_2_O_2_ exposure, mtROS production increased by 4-fold compared with control (*p*<0.01) and pre-treatment with resveratrol or astaxanthin reduced this by 50% (*p*<0.05). H_2_O_2_ exposure reduced GSH/GSSG ratio and this decline was prevented by antioxidants pre-treatment. H_2_O_2_ exposure caused 2.5-fold increase in p21 mRNA expression compared with control (*p*<0.05), while a slight decrease in p21 mRNA expression was observed when cells were pre-treated with resveratrol or astaxanthin. Our results demonstrate that antioxidants, resveratrol, and astaxanthin were able to protect cells from an acute OS. These agents show promise that encourages further research.

## Introduction

Oxidative stress (OS) plays an important role in several respiratory disorders such as asthma, chronic obstructive pulmonary disease (COPD), idiopathic pulmonary fibrosis (IPF), ataxia-telangiectasia (A-T) and respiratory infections [1]. COPD is linked to increased mitochondrial reactive oxygen species (mtROS) production, reduced numbers of mitochondria and decreased intracellular antioxidants [2]. Studies have suggested that antioxidant-based therapy can be used to increase individual’s antioxidant capacity and provide protection from OS [3,4]. Despite increasing preclinical evidence on the use of antioxidants in reducing lung disease in animal models, a lack of clinical evidence for safety and efficacy of antioxidant in preventing or treating lung diseases remains [5].

There are some natural products and food that contains high levels of antioxidants and have antioxidant capacity including lavender essential oil [6], plant or herb extracts [7]. Antioxidant capacity can be scored by oxygen radical absorbance capacity (ORAC). Astaxanthin (3,3’-dihydroxy-β, β’-carotene-4,4’-dione) is a xanthophyll carotenoid produced by algae, bacteria, and fungi, including *Haematococcus pluvialis*, *Chlorella zofingiensis*, *Chlorococcum*, and *Phaffia rhodozyma* [8], and found widely in seafood such as shrimp, lobster, and crab, and especially in their shell portions. Interestingly, Astaxanthin has a very high ORAC score and can bind between the cell membrane. Astaxanthin is a more potent antioxidant than vitamin C [9]. Clinically, astaxanthin has shown multiple benefits including protection against OS and inflammation with high safety and tolerability and it can slow down the age-related functional decline [9]. Astaxanthin has successfully inhibited apoptosis via the ROS-dependent mitochondrial signaling pathway in alveolar epithelial cells type II *in vivo* and *in vitro* [10], and protected cigarette smoke-induced emphysema in mice by activating Nrf2 [11]. Most of the studies investigating the utility of astaxanthin on prevention or treatment of respiratory diseases were conducted on cell lines or *in vivo* rodent models. Thus, there is potential for this area to be explored.

Resveratrol (3,5,4’-trihydroxystilbene) is a natural polyphenol compound that can be found in plants, grapes, berries, and red wine [12]. Many studies have indicated that resveratrol protects against OS and inflammation by enhancing SIRT1 deacetylase activity [13]. SIRT1 plays an important role in regulating OS. Thus, there has been several attempts to boost the function of SIRT1. Polyphenols such as resveratrol, quercetin and catechins are well known antioxidants which enhance SIRT1 deacetylase activity [13]. Resveratrol activated Nrf2 and induced glutathione synthesis in a cigarette smoke-induced oxidative stress model in the human type II alveolar epithelial cell line (A549) [14]. Resveratrol has protected *Staphylococcus aureus*-induced inflammation in human lung epithelial cells cell line (HPAEpiCs) and *in vivo* model [15], and lipopolysaccharides and cigarette smoke-induced COPD rats [16].

The nasal airway epithelium provides the first line of innate immune protection to defend against environmental stressors and infections. A decline in airway epithelial innate immune system functionality is associated with many medical conditions and can result in chronic inflammation and compromised immunity of the lung [17]. Therefore, maintaining the health of the airway epithelium is critical. We have developed a model for studying redox homeostasis in respiratory epithelium using primary nasal epithelial cells grown into a fully differentiated epithelium at the air-liquid interface (ALI) [18]. The aim of this study was to test whether resveratrol and astaxanthin can protect the airway epithelium from H_2_O_2_-induced OS and restore redox homeostasis.

## Material and Methods

Detailed methods have been previously reported [18].

### Cell collection

Healthy non-atopic, non-smoking adult volunteers between the ages of 18 and 65 years were recruited, and primary human nasal epithelial cells (NECs) collected using Rhino-Pro Nasal Curette (Arlington Scientific, UT, USA). Ethics approval: No.#UQ2017000520; HREC61894; UQ2020001742. The nasal scrapings were taken from the inferior turbinate in each nostril and cells were grown as submerged cultures in PneumaCult^™^-Ex Medium (STEMCELL Technologies, BC, Canada) until they reached passage 2.

### Air-liquid interface culture

Cells were seeded (4*10^4^ cells/insert) onto 6.5 mm Transwell^®^ with 0.4 μm Pore Polyester Membrane Inserts (Corning, NY, USA) and differentiated with PneumaCult-ALI Medium (STEMCELL Technologies). A successfully differentiated ALI culture contains basal cells, tight junctions, secretory cells (primarily mucus-secreting goblet cells) and ciliated epithelial cells.

### Prevention of OS with antioxidants

As previously reported [18], H_2_O_2_ was used to induce acute OS in the airway epithelium. As in our previous study [18], we have classified donors as being sensitive or resistant to OS based on the concentration of H_2_O_2_ required to achieve a defined response on epithelial integrity. The donors have different susceptibility to H_2_O_2_, if cells were exposed to the same concentration of H_2_O_2_, we would not be able to investigate potential effects of antioxidants on cells from resistant donors, as they have greater ability to resist H_2_O_2_ exposure. Sensitive donors who are more vulnerable, may benefit from taking antioxidants as a daily supplement to defend against OS.

When the NECs were fully differentiated, cells were treated with 20 μM resveratrol (R5010; Sigma-Aldrich, MI, USA) or astaxanthin (SML0982; Sigma-Aldrich, MI, USA) added to the basal media for 24 hours. NECs were subsequently exposed to 25 mM (sensitive group) or 50 mM (resistant group) H_2_O_2_ in Hanks’ Balanced Salt solution (HBSS; Sigma-Aldrich, MI, USA) in the apical chamber for one hour to induce an acute OS.

### Reagents and assay kits

MitoSOX^™^ Red mitochondrial superoxide indicator (Invitrogen^™^, CA, USA); GSH/GSSG Ratio Detection Assay Kit II (Abcam, Cambridge, UK); Luminescent ATP Detection kit (Abcam); TRIzol^™^ reagent (Invitrogen^™^, CA, USA); RNeasy Mini Kit (Qiagen, Hilden, Germany); High Capacity cDNA Reverse Transcription Kit (Applied Biosystems^™^, CA, USA).

### Quantitative Reverse Transcription PCR (qRT-PCR)

qPCR was performed with Taqman primers (Applied Biosystems^™^, CA, USA): *SIRT1* (Hs01009005_m1) and *CDKN1A* (*p21*; Hs99999142_m1) with FAM dye, and housekeeping gene Eukaryotic 18S rRNA Endogenous Control (4319413E) with VIC dye.

Quantification of gene expression was performed using a ViiA^™^ 7 Real-Time PCR System (Applied Biosystems, MA, USA) and the relative mRNA expression levels were normalized to 18s using the 2^−ΔΔCq^ methods.

### Western blot

Immobilon-P PVDF membrane (Merck KGaA, Darmstadt, Germany) were immunoblotted with primary antibodies: SIRT1 (8469S; Cell Signaling, MA, USA), phosphorylated-SIRT1 (2314S; Cell Signaling), phosphorylated-PARKIN (PA1-4735; Invitrogen^™^), GAPDH (2118S; Cell Signaling) (1:1000); PARKIN (39-0900; Invitrogen^™^) (1:500) and appropriate fluorescent secondary antibodies, anti-rabbit (5366P; Cell Signaling) (1:10,000) or mouse (5257P; Cell Signaling) (1:5,000). The results were detected using the LI-COR Odyssey (BioAgilytix, NC, USA) and quantified by ImageJ software.

### NRF2 nuclear translocation assay

NRF2 nuclear translocation was examined by immunostaining as described previously [19]. NRF2 antibody (1:100) (sc-365949; Santa Cruz Biotechnology, TX, USA); anti-mouse secondary antibody, Alexa Fluor 647 (1:250) (A21235; Invitrogen^™^); Hoechst 33342 nuclei stain (1:10,000). Images were visualized and captured on the Zeiss Confocal LSM 710 (ZEISS, Oberkochen, Germany) and the mean intensity of nuclear NRF2 was quantified by the Image J software.

### Statistical analysis

All the graphs were plotted using GraphPad Prism 9.00 (GraphPad Inc., La Jolla, CA, USA) and were expressed as the mean ± standard deviation.

Kruskal-Wallis rank test with Dunn’s multiple-comparison test was performed to compare different conditions. Two-sample Wilcoxon rank-sum (Mann-Whitney) test was used to compare sensitive and resistant groups. *P*<0.05 was considered to indicate a statistically significant difference.

## Results

### Inhibition of mtROS generation by resveratrol and astaxanthin

**Figure 1.**
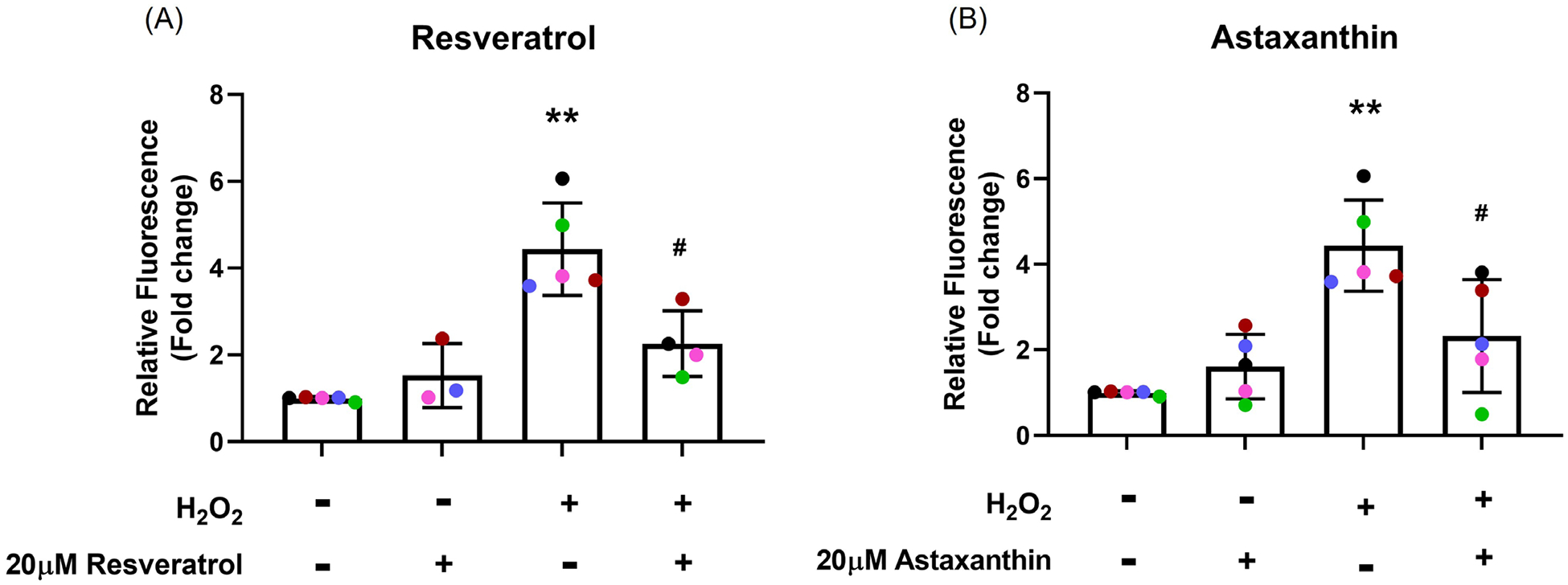
Inhibition of mtROS generation by resveratrol and astaxanthin. NECs were pretreated with antioxidants, resveratrol and astaxanthin, for 24 hours and were subsequently exposed to H_2_O_2_ for one hour. MitoSOX staining was performed, and images were taken by confocal microscopy. The relative fluorescence of MitoSOX signals was quantified; resveratrol **(A)** and astaxanthin **(B)**. Data presented as mean ± SD (n=5; * *p*<0.05; ** *p*<0.01, compared with control; ^#^ *p*<0.05, compared with H_2_O_2_). A single color was used in all figures to represent a single donor.

mtROS can be seen in the cytoplasm (merged panel) with H_2_O_2_ exposure and this signal was reduced in antioxidant-treated cells (Figure S1). Compared with control, cells exposed to H_2_O_2_ showed a 4-fold increase in mtROS production (*p*<0.001; Figure 1). Pre-treatment with resveratrol or astaxanthin reduced this by 50% (*p*=0.044, *p*=0.028, respectively; Figures 1A and 1B).

### Resveratrol and astaxanthin restored GSH/GSSG ratio

**Figure 2.**
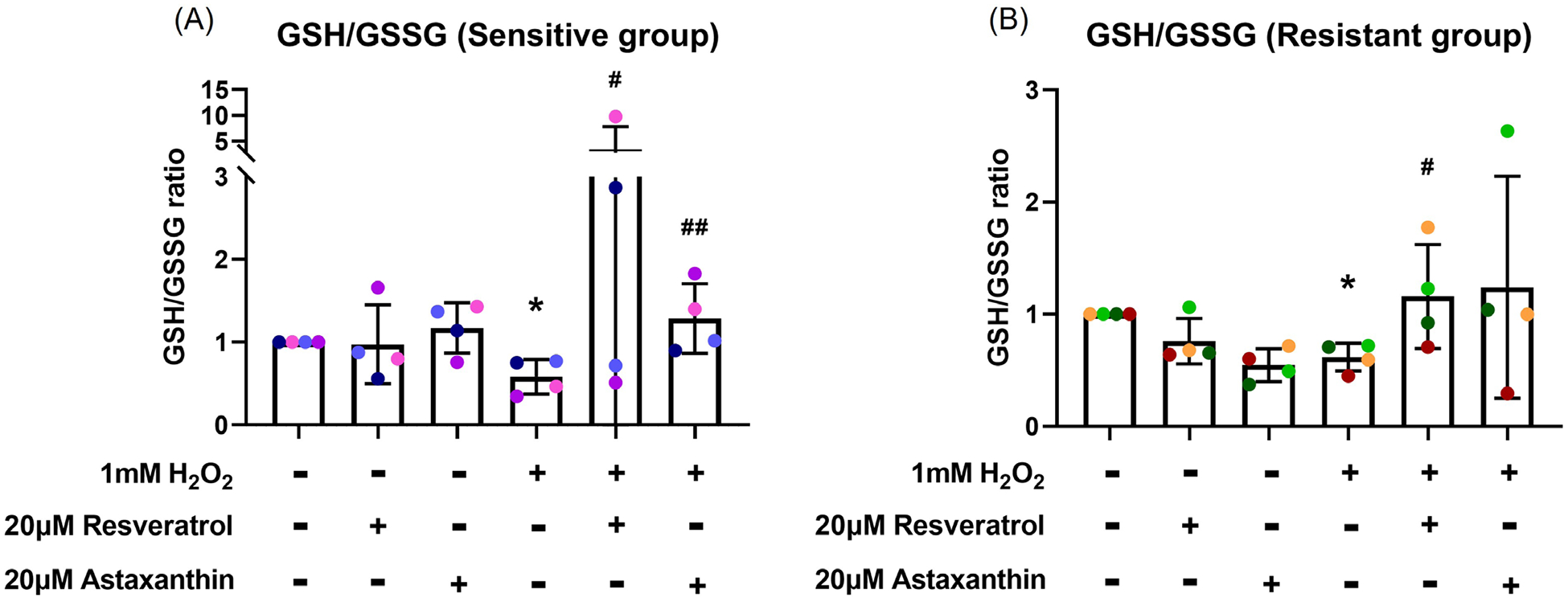
Resveratrol and astaxanthin restored H_2_O_2_-induced GSH/GSSG ratio reduction. Following three-hour H_2_O_2_ basal exposure, GSH/GSSG ratio decreased. This was reversed by antioxidants in the sensitive **(A)**, and resistant **(B)** groups. Data presented as mean ± SD (n=8; * *p*<0.05, compared with control; ^#^ *p*<0.05; ^##^ *p*<0.01, compared with H_2_O_2_). A single color was used in all figures to represent a single donor.

GSH/GSSG assay was performed to examine whether resveratrol and astaxanthin can prevent the H_2_O_2_ reduced GSH/GSSG ratio. Following three hours of 1 mM H_2_O_2_ basal exposure, there was a decrease in GSH/GSSG ratio in both sensitive (*p*=0.032; Figure 2A) and resistant (*p*=0.028; Figure 2B) groups with more reduction observed in the sensitive group compared to the resistant group. Pre-treatment with resveratrol had a marked effect in increasing the GSH/GSSG ratio in 2 sensitive donors, with less impressive effects on the other two. On grouped data, resveratrol and astaxanthin prevented GSH/GSSG ratio reduction in both sensitive (*p*=0.028, *p*=0.005, respectively; Figure 2A) and resistant (*p*=0.032, *p*=0.080, respectively; Figure 2B) groups. Overall, astaxanthin was more effective than resveratrol in both groups. Resveratrol pretreatment prevented GSH/GSSG reduction to the same level as control or more (GSH/GSSG ratio ≥ 1) in 2 sensitive donors and 2 resistant donors. On the other hand, astaxanthin pretreatment prevented the reduction in 3 sensitive donors and 3 resistant donors. However, there was no statistical difference between the effectiveness of resveratrol and astaxanthin.

### Resveratrol and astaxanthin did not prevent H_2_O_2_-induced reduction in ATP production

ATP production was measured to examine whether resveratrol and astaxanthin could prevent H_2_O_2_-induced reduced cellular ATP production. Following one-hour H_2_O_2_ exposure, there was a decrease in cellular ATP in both sensitive (*p*=0.002; Figure S2A) and resistant (*p*=0.036; Figure S2B) groups. Pre-treatment with resveratrol and astaxanthin did not prevent the fall in ATP production induced by H_2_O_2_ exposure in both sensitive (*p*=0.450, *p*=0.460, respectively; Figure S2A) and resistant (*p*=0.183, *p*=0.291, respectively; Figure S2B) groups.

### Resveratrol and astaxanthin inhibited H_2_O_2_-induced upregulation of *p21* mRNA expression but did not affect *SIRT1* mRNA expression

**Figure 3.**
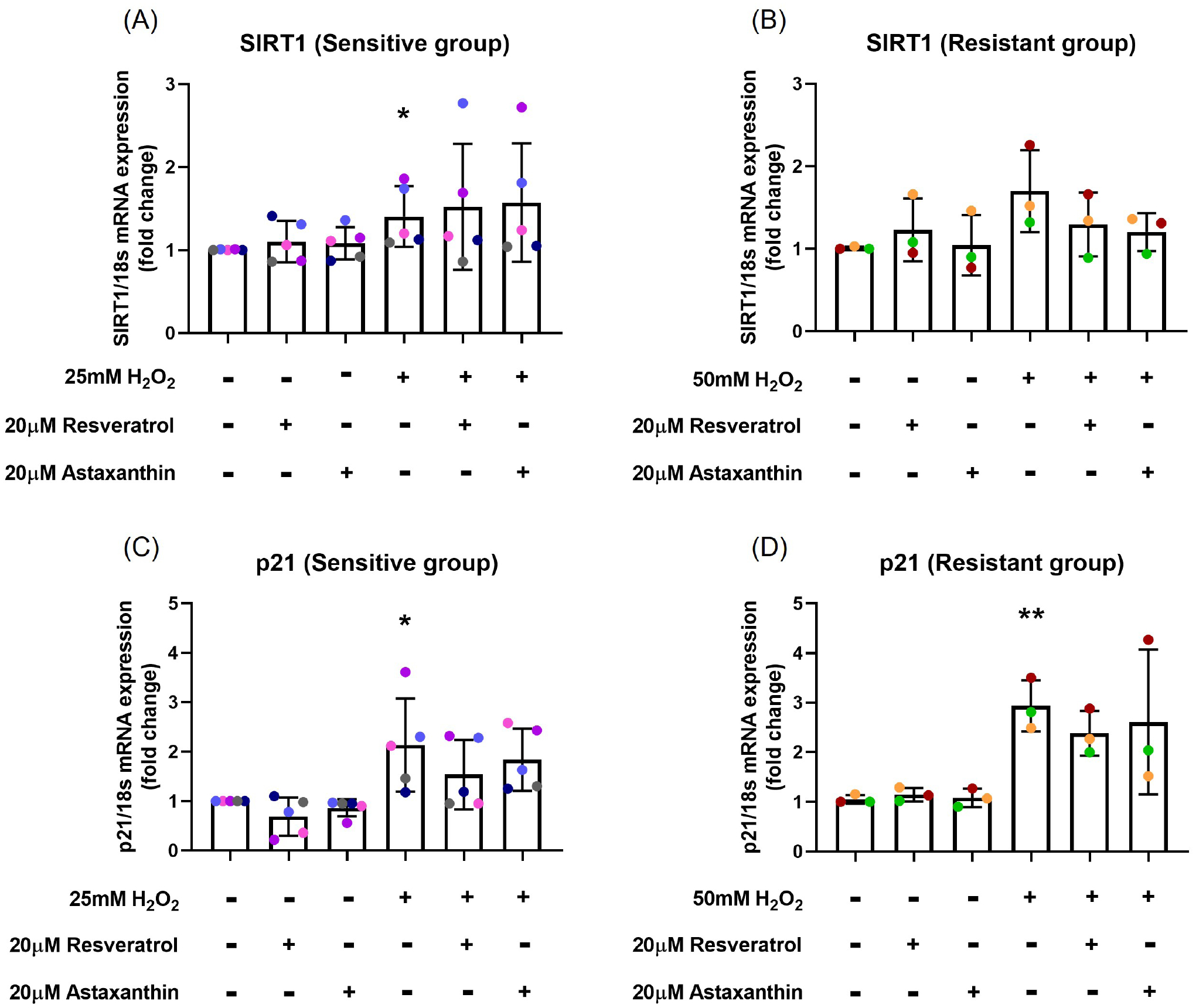
Resveratrol and astaxanthin inhibited *p21* mRNA expression but did not affect *SIRT1* mRNA expression. H_2_O_2_ exposure increased *SIRT1* mRNA expression about 1.4-fold in the sensitive **(A)** and 1.7-fold in the resistant **(B)** groups, while there was no significant difference in *SIRT1* mRNA expression with resveratrol or astaxanthin treatment **(A, B)**. H_2_O_2_ exposure caused 2.1-fold increase in *p21* mRNA expression in the sensitive **(C)** and 2.9-fold increase in the resistant **(D)** groups, while a slight decrease in *p21* mRNA expression was observed when cells were pre-treated with resveratrol or astaxanthin **(C)**. Data presented as mean ± SD (n=8; *p<0.05; ** *p*<0.01, compared with control). A single color was used in all figures to represent a single donor.

To examine whether resveratrol and astaxanthin were able to inhibit the upregulation of H_2_O_2_-induced *p21* and *SIRT1* mRNA expression, RT-PCR was performed. H_2_O_2_ exposure increased *SIRT1* mRNA expression about 1.4-fold (*p*=0.04; Figure 3A) in the sensitive group and 1.7-fold (*p*=0.07; Figure 3B) in the resistant group, while there was no significant difference in *SIRT1* mRNA expression with resveratrol (*p*=0.762, *p*=0.329, respectively; Figures 3A and 3B) or astaxanthin (*p*=0.652, *p*=0.190, respectively; Figures 3A and 3B) pre-treatment in both sensitive and resistant groups.

H_2_O_2_ exposure caused 2.1-fold increase in *p21* mRNA expression compared with control (*p*=0.03; Figure 3C) in the sensitive group and 2.9-fold increase in the resistant group (*p*=0.003; Figure 3D). Although, there was no statistical differences, a 29% decrease in *p21* mRNA expression was observed when cells were pre-treated with resveratrol (*p*=0.291; Figure 3C) in the sensitive group. In individual level, resveratrol was more effective than astaxanthin in some donors (pink and red donors; Figures 3C and 3D), while for others (blue and orange donors; Figures 3C and 3D) astaxanthin worked better on preventing H_2_O_2_-induced *p21* mRNA upregulation.

### H_2_O_2_ exposure, resveratrol and astaxanthin increased SIRT1, HO-1 protein level and NRF2 translocation

To investigate whether the antioxidants, resveratrol and astaxanthin, enhanced the SIRT1-mediated antioxidant signaling pathway and influenced the mitophagy pathway, SIRT1 and PARKIN phosphorylation, HO-1 protein level and NRF2 nuclear translocation were measured.

**Figure 4.**
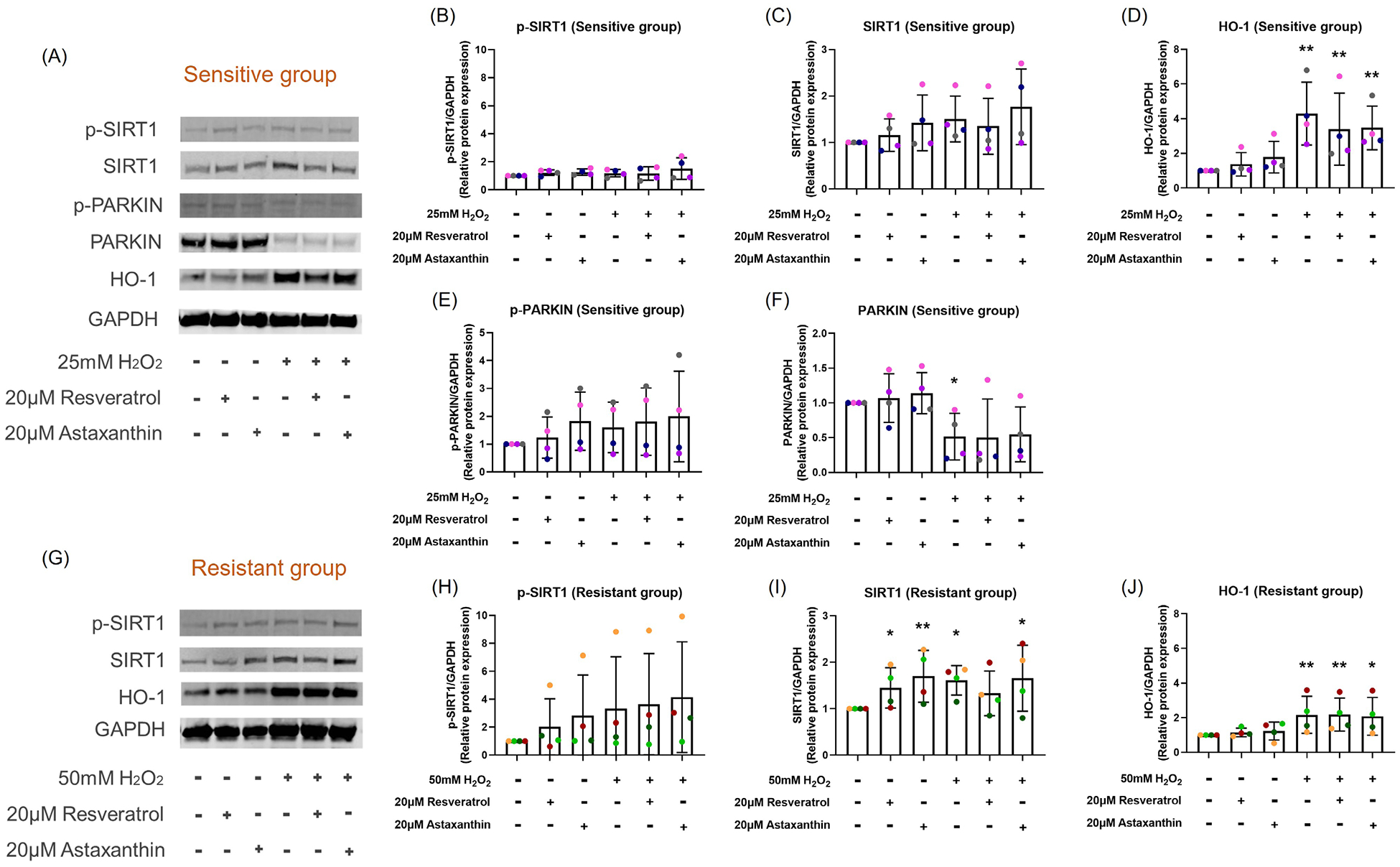
Resveratrol or astaxanthin did not affect H2O2-induced SIRT1 and HO-1 protein expression levels. Images are shown from representative sensitive **(A)** and resistant **(G)** donors. H2O2 exposure increased total SIRT1 protein expression and HO-1 protein expression in both sensitive **(A, C, D)** and resistant **(G, I, J)** groups. These increases were not affected by resveratrol or astaxanthin pre-treatment. Resveratrol and astaxanthin alone increased SIRT1 total protein level as much as H2O2 exposure in the resistant group **(I)**. There was no significant difference in the levels of phosphorylated SIRT1 observed in either group **(B, H)**. H2O2 exposure reduced total PARKIN protein expression that was not prevented by resveratrol or astaxanthin pre-treatment in the sensitive group **(A, F)**. Data presented as mean ± SD (n=8; *p<0.05; ** *p*<0.01, compared with control). A single color was used in all figures to represent a single donor

At 2-hours post H_2_O_2_ exposure, an increase in total SIRT1 protein levels in the resistant (*p*=0.018; Figures 4G and 4I) group was observed but not in the sensitive (*p*=0.060; Figures 4A and 4C) group. However, there was no significant difference in the levels of phosphorylated SIRT1 observed in either group (Figure 4B and 4H). Resveratrol and astaxanthin treatments alone caused an increase in SIRT1 total protein level in the resistant group (*p*=0.049, *p*=0.008, respectively; Figure 4I), but not in the sensitive group (*p*=0.401, *p*=0.212, respectively; Figure 4C).

HO-1 protein level was upregulated by 24-hour H_2_O_2_ post exposure in both sensitive (*p*<0.001; Figure 4D) and resistant (*p*=0.004; Figure 4J) groups with no difference in resveratrol (*p*=0.212, *p*=0.490, respectively; Figures 4D and 4J) or astaxanthin (*p*=0.344, *p*=0.344, respectively; Figures 4D and 4J) pre-treatment. The sensitive donors increased HO-1 (4.3-fold) to a much greater degree than the resistant donors (2.2-fold) following H_2_O_2_ exposure. Astaxanthin treatment alone increased HO-1 protein expression by 1.8-fold in the sensitive group (*p*=0.055), and a slight increase in the resistant group (*p*=0.084).

PARKIN phosphorylation and total protein level were only measured in the sensitive group, since H_2_O_2_ exposure had no effect on the resistant donors as shown in previous study [18]. H_2_O_2_ exposure did not change the phosphorylated PARKIN level (*p*=0.274; Figure 4E) but decreased total PARKIN protein level (*p*=0.042; Figure 4F) with no change with resveratrol or astaxanthin treatment (*p*=0.460, *p*=0.326, respectively; Figures 4E and 4F) at 24-hour post exposure.

**Figure 5.**
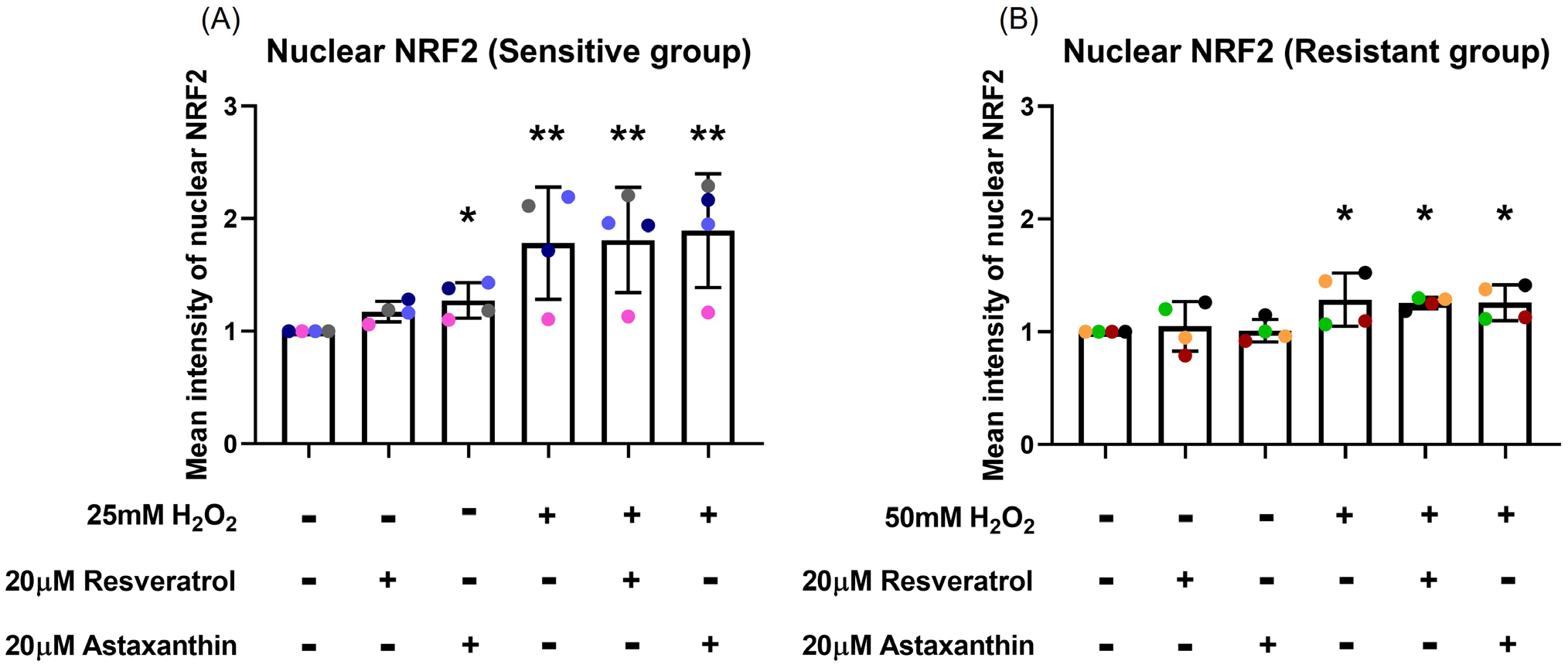
Resveratrol and astaxanthin did not affect H_2_O_2_-induced nuclear NRF2 translocation. Nuclear NRF2 increased by 4-hour post H_2_O_2_ exposure; this was not affected by resveratrol or astaxanthin pre-treatment in both sensitive **(A)** and resistant **(B)** groups. Data presented as mean ± SD (n=8; *p<0.05; ** *p*<0.01, compared with control). A single color was used in all figures to represent a single donor.

At 4-hour post H_2_O_2_ exposure, nuclear NRF2 increased in the sensitive group (*p*=0.003; Figures 5A). This increase was not prevented by resveratrol or astaxanthin pre-treatment (*p*=0.460, *p*=0.344, respectively; Figure 5A). NRF2 translocation to the nucleus was less prominent in the resistant group, with only slight increases in nuclear NRF2 following H_2_O_2_ exposure (*p*=0.018; Figure 5B). Again, resveratrol or astaxanthin pre-treatment did not influence nuclear translocation of NRF2 (*p*=0.440, *p*=0.500, respectively; Figure 5B). Resveratrol and astaxanthin without H_2_O_2_ increased nuclear NRF2 in the sensitive group (*p*=0.073, *p*=0.036, respectively; Figure 5A). Images of a representative sensitive individual (Figure S3A) and of a representative resistant individual (Figure S3B) are shown in the online supplement.

## Discussion

Increased ROS in the lungs can lead to pulmonary fibrosis, COPD, asthma, acute respiratory distress syndrome, and lung cancer [20]. Antioxidant therapy is one of the encouraging treatments for intervening in an exposure-induced redox imbalance [21]. The objective of this study was to test the utility of the antioxidants, resveratrol and astaxanthin, on H_2_O_2_-induced oxidative stress on well-differentiated epithelium cultured at ALI.

The impact of various antioxidants on respiratory health have been studied and some of them are available as drugs or food supplements [1]. However, none of these dietary antioxidants have shown strong evidence for attenuating OS induced by oxidant exposure. Several studies have shown that the well-known antioxidant N-acetylcysteine (NAC) can prevent OS within human airway cells [22,23]. Unfortunately, there is some evidence to indicate that NAC has poor cell permeability [24] and acts by neutralizing free radicals outside cells [25]. Resveratrol and astaxanthin are nutritional compounds with high safety and tolerability [9]. Both have antioxidant capacity and can protect against OS and inflammation. In the present study, we observed that resveratrol and astaxanthin prevented H_2_O_2_-induced mtROS generation. This is in agreement with previous studies showing that resveratrol attenuated high glucose-induced mtROS generation in coronary arterial endothelial cells [26] and astaxanthin inhibited homocysteine-induced mtROS production in H9c2 rat myocardial cells [27]. Resveratrol can inhibit complex I activity in the mitochondrial respiratory chain, one of the major sources of ROS [28]. Astaxanthin increased the antioxidant enzymes SOD2 and CAT concentrations in the absence of stimuli in Wistar male rats and pufferfish [29,30]. Increasing the cellular levels of antioxidant enzymes may be the mechanism by which astaxanthin acts in preventing H_2_O_2_-induced mtROS generation in our studies. In addition, astaxanthin can accumulate in mitochondria [31], which may advance removal of mtROS.

Oxidant exposure results in oxidation of GSH to GSSG, reducing the GSH/GSSG ratio in the cells. Resveratrol and astaxanthin prevented H_2_O_2_-induced reduction in GSH/GSSG ratio in both sensitive and resistant groups, which indicates that resveratrol and astaxanthin can restore redox homeostasis. Two of the sensitive individuals showed marked increases in GSH/GSSG ratio following resveratrol treatment in the presence of H_2_O_2_. This might suggest that resveratrol is a good candidate for precision therapy in those two donors.

H_2_O_2_-induced reduction in ATP production was not prevented by resveratrol or astaxanthin pre-treatment. Resveratrol can inhibit ATPase activity by binding to γ subunit F_1_ site of ATP synthase. ATPase is a catalysis enzyme involve in ATP synthesis [32]. Although, resveratrol itself did not cause a significant drop in ATP production, it did not prevent the reduction of ATP levels following H_2_O_2_ exposure. This result is consistent with a study in primary human periodontal ligament fibroblasts that a resveratrol metabolite, piceatannol, did not provide protection from H_2_O_2_ exposure causing ATP reduction [33]. In an *in vivo* study in mice, resveratrol restored oxidant exposure reduced ATP production in the hippocampus [34]. The relevance of this study to the human respiratory epithelium is unknown. Our studies used primary human respiratory epithelial cells grown in ALI culture to a fully differentiated airway epithelium and may be more relevant to human environmental exposures. Astaxanthin has been shown to increase subunit α of F_1_ATP synthase, subunit *b* and *c* of F_0_ATP synthase concentration following isoproterenol-induced OS in Wistar male rats that enhances ATP production [29]. A mouse neural progenitor cell study showed that astaxanthin restored H_2_O_2_-reduced ATP production [35]. Both studies are in contrast with our result, this might be because our samples were from humans and those studies were in mice.

When cells are exposed to oxidants the stress resistance pathway is activated. Effects of oxidant exposure include direct damage to mtDNA/DNA, activation of the p53-p21 apoptotic pathway [2], the antioxidant pathways SIRT1-FOXO3 [36] and SIRT1-NRF2-HO-1 [37], as well as the SIRT1-NRF2-PINK1/PARKIN mitophagy pathway through mitochondria [38]. The Sirtuin family (SIRTs) of proteins is closely associated with mitochondrial integrity and stress tolerance mediation [39]. NRF2 is a stress-response transcription factor associated with ROS homeostasis by regulating and encoding antioxidant and detoxifying enzymes such as SOD, GPx and HO-1 [40, 41]. NRF2 is known to be a key mediator of SIRT1-related metabolic effects and SIRT1 activates NRF2 nuclear translocation [42]. Our results showed that resveratrol and astaxanthin slightly inhibited H_2_O_2_-induced *p21* mRNA upregulation, thus inhibiting activation of an apoptosis pathway. H_2_O_2_ exposure caused an increase in *SIRT1* mRNA expression, SIRT1 and HO-1 protein expression, and NRF2 nuclear translocation, that was not affected by resveratrol or astaxanthin pre-treatment. These results differ from a study in mouse liver sinusoidal endothelial cells showing that an herbal antioxidant increased H_2_O_2_-induced Nrf2 and HO-1 protein expression [43]. This again highlights the difference between studies conducted in mice and our studies conducted in human cells. Interestingly, resveratrol and astaxanthin alone increased total SIRT1 protein level and nuclear NRF2. In addition, astaxanthin alone increased HO-1 protein expression. These data may suggest that SIRT1 is the target of resveratrol and astaxanthin. Several studies demonstrated that resveratrol could enhance SIRT1 activity [44]. Astaxanthin can increase SOD2 and CAT antioxidant enzyme concentrations and this can be mediated by SIRT1 pathways [45]. One study showed that astaxanthin itself increased in Nrf2 and HO-1 protein expression in C57BL/6 mice [11], which is similar to our results. These suggested that resveratrol or astaxanthin supplements can enhance individual’s antioxidant capacity. Mitophagy can decrease intracellular ROS by removing damaged mitochondria, stimulating creation of new healthy mitochondria. In our studies, H_2_O_2_ exposure reduced total PARKIN protein levels. This was not prevented by pre-treatment with resveratrol or astaxanthin. This suggested that resveratrol and astaxanthin protected the airway epithelium from the H_2_O_2_ induced OS was not via mitophagy pathway.

## Conclusions

**Figure 6.**
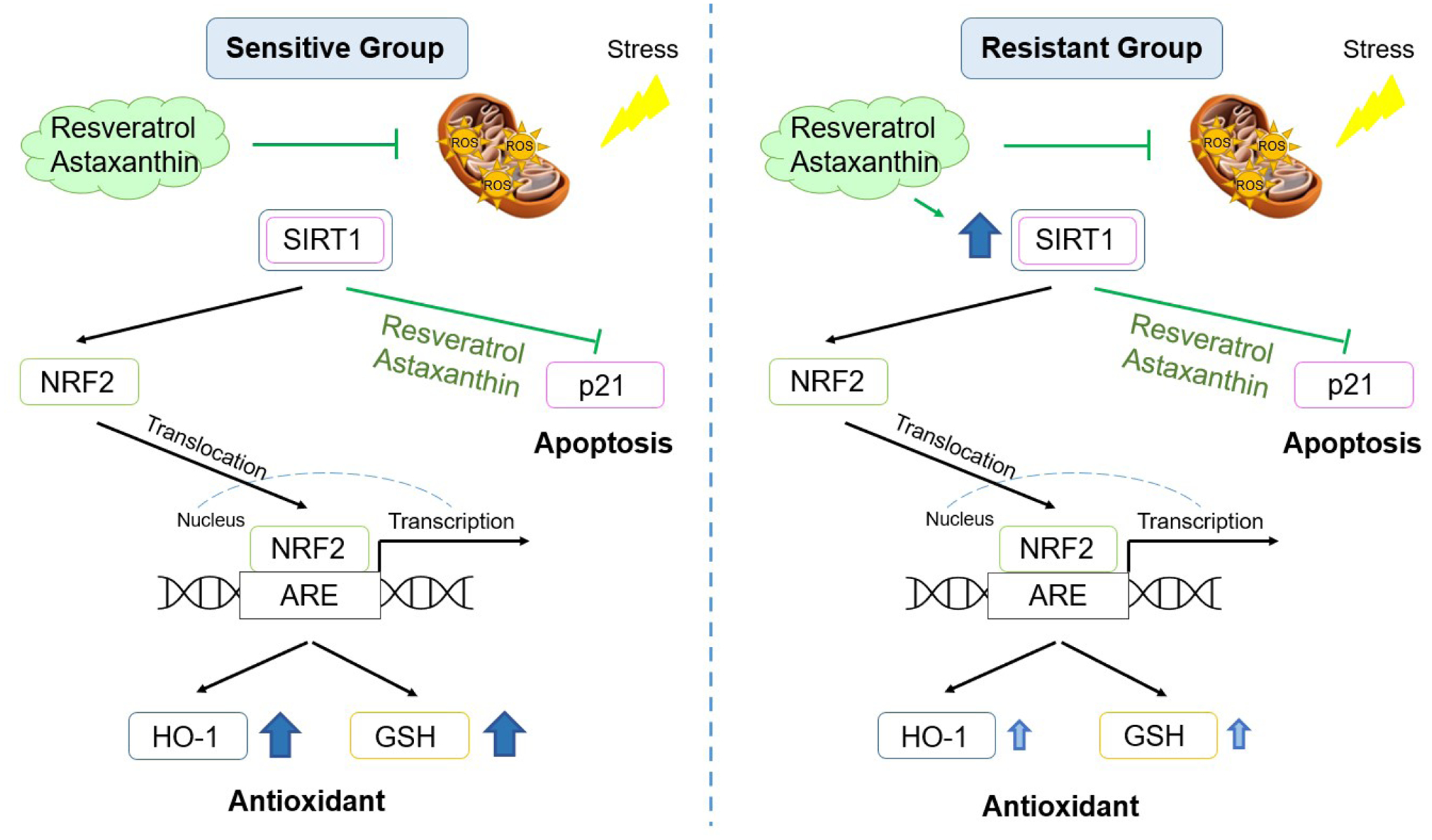
Signaling pathway diagram in the sensitive and resistant groups with resveratrol and astaxanthin. Green: inhibited by resveratrol or astaxanthin; Dark blue: significantly increased; Light blue: slightly increased.

The utility of the antioxidants, resveratrol and astaxanthin, in preventing H_2_O_2_ induced OS was examined. Our results demonstrated that resveratrol and astaxanthin protected cells from an acute oxidant exposure by inhibiting mtROS generation and promoting SIRT1-NRF2 antioxidant pathway. They also prevented reduction of the GSH/GSSG ratio, preserving redox homeostasis. A signaling pathway diagram summarizing the results reported in this study is shown in Figure 6.

## Supplementary Material

JCS-22-084_Supplementary File
