# Supplementary material for: Resveratrol and Astaxanthin Protect Primary Human Nasal Epithelial Cells Cultured at an Air-liquid Interface from an Acute Oxidant Exposure
Source: J Cell Signal. Author manuscript; Available in PMC 2023 Feb 9. (PMC9910318; doi:10.33696/signaling.3.084)
Supplement: JCS-22-084_Supplementary File [file NIHMS1869070-supplement-JCS-22-084_Supplementary_File.pdf]

## Supplementary Figures

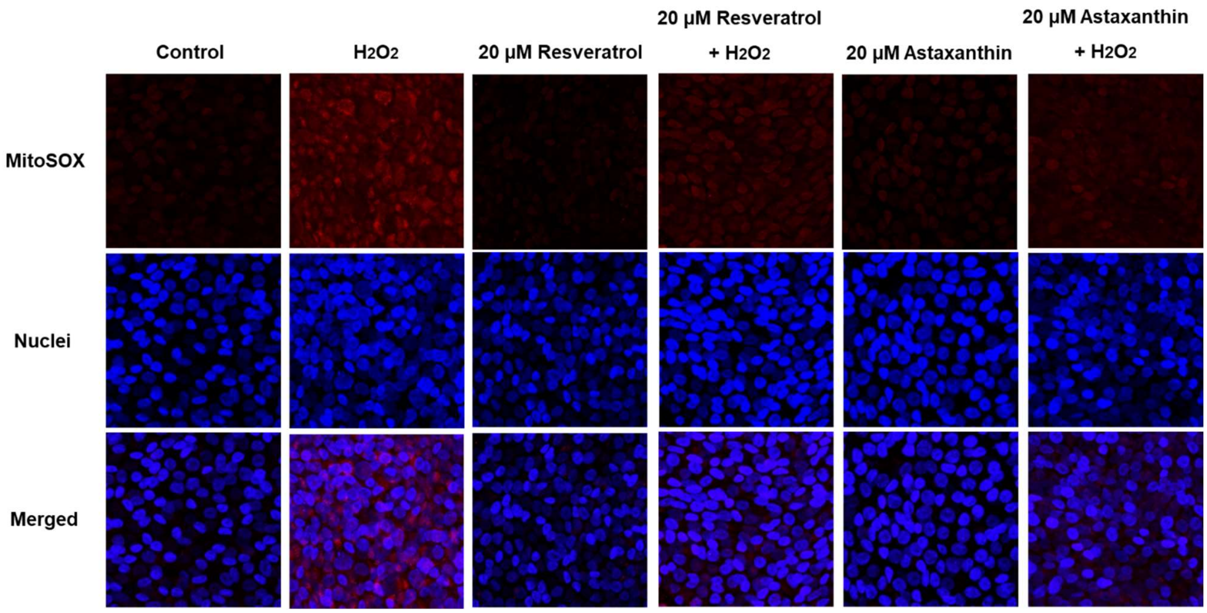


**Figure S1. Inhibition of mtROS generation by resveratrol and astaxanthin.** NECs were pretreated with antioxidants, resveratrol and astaxanthin, for 24 hours and were subsequently exposed to $H_2O_2$ for one hour. MitoSOX staining was performed, and images were taken by confocal microscopy (Red: mtROS; blue: nuclei.), mtROS can be seen in the cytoplasm (merged panel) with $H_2O_2$ exposure and this signal was reduced in antioxidant-treated cells.

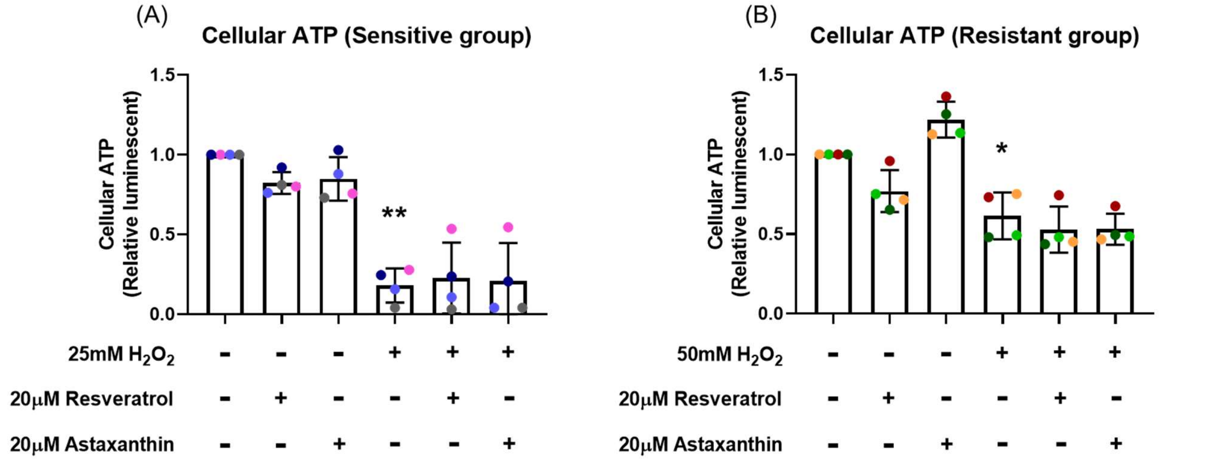


**Figure S2. Resveratrol and astaxanthin could not recover $H_2O_2$-induced ATP reduction.** A decrease in cellular ATP levels following one-hour $H_2O_2$ exposure was observed. ATP levels were not rescued following the addition of antioxidants in the sensitive

(A), and resistant (B) groups. Data presented as mean ± SD (n=8; * $p$<0.05, ** $p$<0.01 compared with control). A single color was used in all figures to represent a single donor.

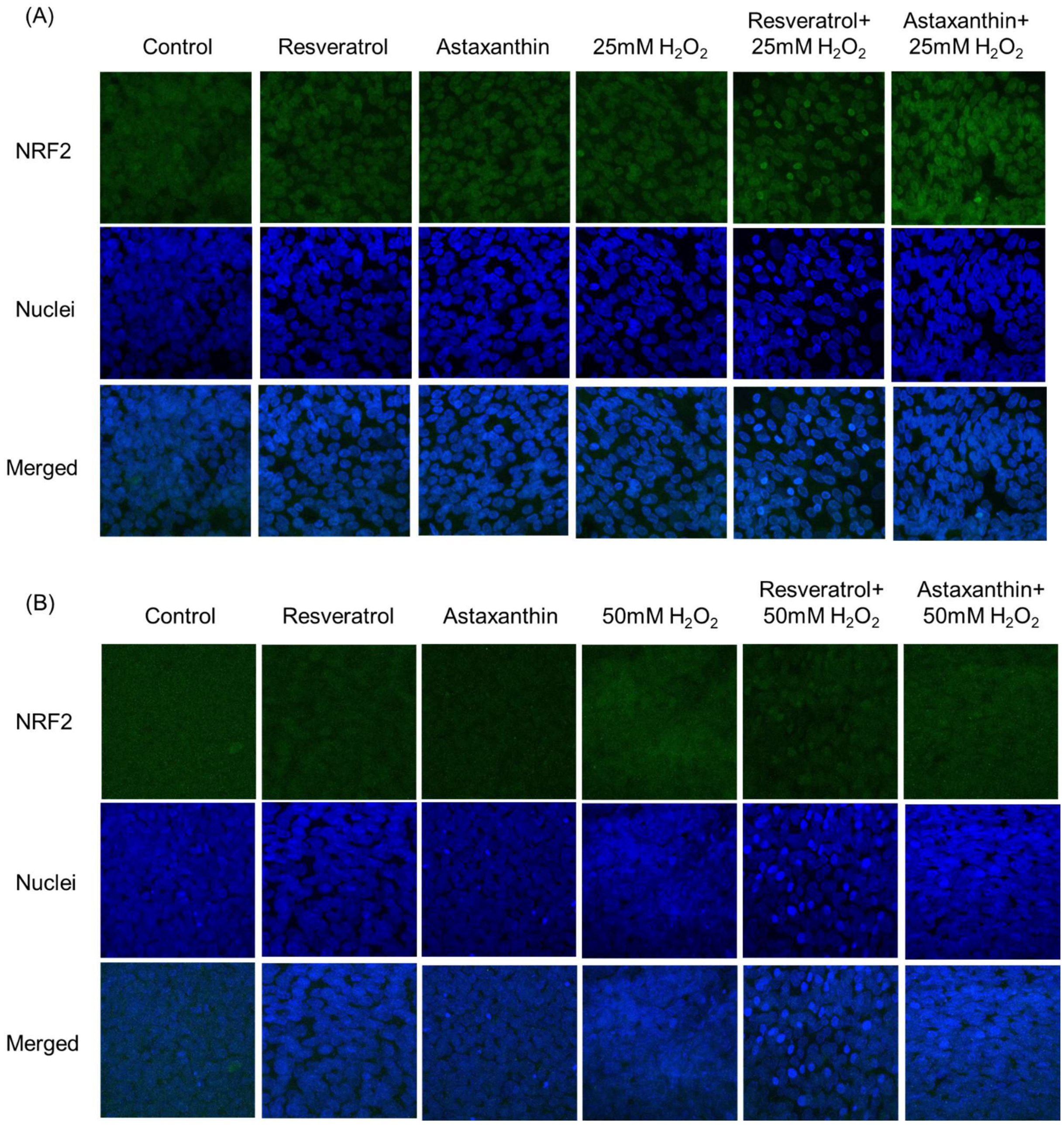


**Figure S3. Resveratrol and astaxanthin did not affect $H_2O_2$-induced nuclear NRF2 translocation.** Images of a representative sensitive individual (A) and of a representative resistant individual (B). Nuclear NRF2 increased by 4-hour post $H_2O_2$ exposure; this was not prevented or improved by resveratrol or astaxanthin pre-treatment.
